# Environmental predictors for the restoration of a critically endangered coral, *Acropora palmata*, along the Florida reef tract

**DOI:** 10.1371/journal.pone.0296485

**Published:** 2024-01-02

**Authors:** Raymond B. Banister, T. Shay Viehman, Stephanie Schopmeyer, Robert van Woesik

**Affiliations:** 1 Institute for Global Ecology, Florida Institute of Technology, Melbourne, FL, United States of America; 2 National Centers for Coastal Ocean Science, National Ocean Service, National Oceanic and Atmospheric Administration, Beaufort, NC, United States of America; 3 Florida Fish and Wildlife, Fish and Wildlife Research Initiative, St. Petersburg, FL, United States of America; MARE – Marine and Environmental Sciences Centre, PORTUGAL

## Abstract

The population decline and lack of natural recovery of multiple coral species along the Florida reef tract have instigated the expanding application of coral restoration and conservation efforts. Few studies, however, have determined the optimal locations for the survival of outplanted coral colonies from restoration nurseries. This study predicts the optimal locations for *Acropora palmata* colonies along the Florida reef tract using a boosted-regression-tree model to examine the relationships between the occurrence of wild *A*. *palmata* and ten environmental variables. Our model results predicted *A*. *palmata* was most likely to occur in shallow reef habitats with (i) generally low mean chlorophyll-a concentrations (< 1 mg m^-3^), (ii) moderate fetch (3 kJ m^-2^), (iii) salinities between 20 and 37.5 ppt, (iv) temperatures between 20 and 32°C, (vi) low mean concentrations of total nitrogen (0.16 ppm), and (iv) irradiance between 26.5 and 53.5 mol m^−2^ s^−1^. The most suitable habitats for *A*. *palmata* were disproportionately allocated to reefs in Biscayne Bay, the Upper Keys, the western-lower Florida Keys, the Marquesas, and the Dry Tortugas. The middle Florida Keys had unfavorable environmental conditions for *A*. *palmata* habitat. Results from this study inform where *A*. *palmata*, outplanted as part of restoration and conservation efforts, would have suitable environmental conditions to persist over time. This study also provides decision-making support for management focused on the conservation and restoration of the endangered species *A*. *palmata* along the Florida reef tract.

## Introduction

Before the 1970s, *Acropora palmata* was a ubiquitous coral along the Florida reef tract [1] where it dominated reef crests and played a critical role in reef-building [1–5]. Since the 1970s, however, the *A*. *palmata* population has drastically declined at an unprecedented rate, primarily because of disease and thermal stress [6, 7]. Early disease outbreaks resulted in the decline of over 95% of *A*. *palmata* in both the Caribbean [6–8] and Florida [7, 9–11]. Over the past four decades, the continued decline of *A*. *palmata* has led to the species being listed as ‘threatened’ under the U.S. Endangered Species Act [12] in 2006 and ‘critically endangered’ under the World Conservation Union (IUCN) Red List in 2008 [13]. Today, *A*. *palmata* populations continue to decline and recruitment rates remain low [8, 10, 14–16].

Drastic declines in the acroporid populations have inspired multiple agencies to attempt to restore the depauperate *Acropora* populations in Florida [17–23]. Restoration of *Acropora cervicornis* and *A*. *palmata* is aided by the rapid growth rates of both species [24, 25]. They are also commonly grown in nurseries because of their ability to reproduce asexually through fragmentation [26]. Restoration of corals has recently blossomed into a multidisciplinary effort, stemming from (i) selective breeding and genetic engineering [27], (ii) optimizing outplant techniques [28], and (iii) selecting the best habitats and geographical localities to enhance the survival of outplanted nursery-reared coral colonies [29–31].

With the rapidly changing environmental conditions worldwide, the increasing prevalence of diseases, and the ongoing thermal-stress events, we can not assume that where a species was located in the distant past is a useful predictor of where that species will be located in the present and future [32]. Optimal locations to outplant nursey-reared coral colonies, instead, have been determined using species distribution models [29, 31], which are invaluable in predicting the fundamental niche of a species, predicting species occurrences, and aiding restoration and conservation efforts [33].

Species-distribution models predict the geographical niche space of a species by examining the locations where a species is present and absent under different environmental conditions. This is done by identifying the geographical coordinate data that determines where the species of interest is present and absent and by identifying environmental conditions that potentially describe the niche space of the species. From these data, models are constructed to determine the theoretical-environmental space for the species, which is then translocated back onto geographic space to determine where a species is best supported, given the suite of environmental conditions.

Species-distribution models can also utilize mechanistic functions that can include the occurrence of physical barriers, such as rivers, or dispersal kernels, to refine the predictions of species occurrence [33–36]. Species-distribution models, however, can have drawbacks, particularly when used on geographically ubiquitous species, as they can underestimate vulnerability under climate change when local adaptation and hydrodynamic barriers are not identified [37]. Modeling rare species is also challenging because low occurrences, driven by recent disturbances, prevent some species from occupying their entire fundamental niche space [31, 38]. Not occupying their full niche space can lead to models with a low accuracy at predicting where the species should be located (i.e., true positives). Indeed, recent species-distribution models of coral with low densities, both at local and regional scales, show low sensitivity but high specificity [31]. Low-sensitivity models are inaccurate at predicting true positives, where a species is likely to occur, whereas high-specificity models accurately estimate true negatives, where a species is not likely to occur [39, 40]. While we need information on sensitivity and specificity, high-sensitivity models are needed to facilitate restoration efforts. Additionally, because disturbed populations, with low and patchy densities, do not fully utilize the full extent of a species’ fundamental niche [41], such populations often lead to underestimates of geographical occupancy [31]. Overcoming these challenges for endangered species, with low occurrences, is essential for constructing accurate species-distribution models and aiding coral restoration.

To best create a high-sensitivity species-distribution model, we collated the most extensive geographical data available on *Acropora palmata* dating back to 1999, with 28.4% of the study sites showing occupancy at some point in recent history. In addition, we compiled data for a suite of environmental variables that are known to influence acroporids, including water temperature and irradiance [42, 43], nutrient concentrations [44], ocean productivity [45], salinity [46], and fetch [47]. The objective of our study was to develop a species-distribution model that predicts the optimal locations for *A*. *palmata* colonies along the Florida reef tract to indicate which locations would be most conducive to the survival of outplanted nursery-reared colonies. Accurately determining the most suitable habitat for coral restoration is critical for initiatives such as Mission: Iconic Reefs, which is dedicating resources towards outplanting hundreds of thousands of coral colonies along the Florida reef tract from 2022 to 2027 [48]. This model will provide decision-making support for the restoration and ultimate conservation of the critically endangered *A*. *palmata* corals along the Florida reef tract.

## Methods

### Biological and environmental data

**Fig 1:**
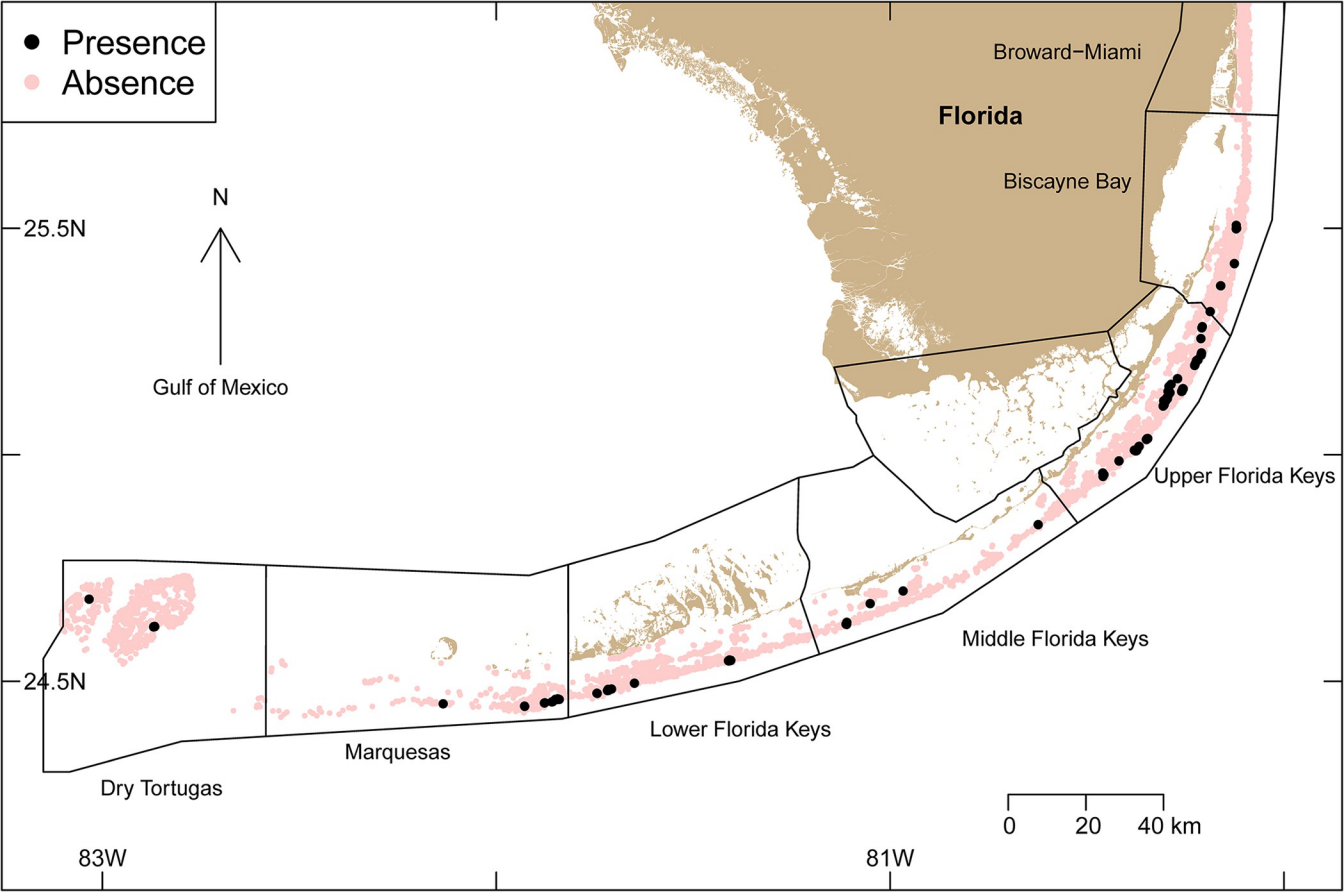
A map depicting the geographic locations of the presence (black dots) and absence (pink shading) of *Acropora palmata* from 25,436 sites sampled between 1999 and 2019 along the Florida reef tract, USA. *A*. *palmata* was present at 7,214 sites (i.e., at 28.4% of the total sites) and absent at 18,222 sites (i.e., at 71.6% of the total sites). These data were obtained from the following five long-term monitoring programs (Table A in S1 File): (i) Florida Fish and Wildlife Conservation Commission’s (FWC) Coral Reef Evaluation and Monitoring Project, (ii) FWC Disturbance Monitoring Program, (iii) *Acropora*-specific monitoring by FWC and the National Oceanic and Atmospheric Administration (NOAA), (iv) NOAA’s National Coral Reef Monitoring Program, and (v) the Sanctuary Coral Reef Ecosystem Assessment and Monitoring Program.

Presence and absence data on *Acropora palmata* were collated from 25,436 study sites along the Florida reef tract from Miami-Dade County to the Dry Tortugas (sampled between 1999 and 2019) where *A*. *palmata* was recorded as present at 7,214 sites (i.e., at 28.4%) of those sites (Fig 1). The data were obtained from the following five long-term monitoring programs (Table A in S1 File): (i) Florida Fish and Wildlife Conservation Commission’s (FWC) Coral Reef Evaluation and Monitoring Project, (ii) FWC Disturbance Monitoring Program, (iii) *Acropora*-specific monitoring by FWC and the National Oceanic and Atmospheric Administration (NOAA), (iv) NOAA’s National Coral Reef Monitoring Program, and (v) the Sanctuary Coral Reef Ecosystem Assessment and Monitoring Program. Programs vary between repeated surveys at fixed sites and stratified random sites; all monitoring was diver-based. When *A*. *palmata* was present at a fixed site at any time between 1999 and 2019, the site was included as present.

The study assessed which environmental conditions were most useful for predicting present-day optimal conditions for *Acropora palmata* along the Florida reef tract. As the niche space of a coral is frequently a consequence of exposure to extremes [37] we focused on the maximum and minimum values of temperature, salinity, and irradiance at a site, mean chlorophyll-a, and total nitrogen and phosphate concentrations. We were also interested in examining the relationship between the presence of *Acropora palmata* and fetch, as a proxy of exposure to storm waves. The near-substrate total nitrogen and total phosphate concentrations (ppm), and salinity (ppt) were acquired from the Southeast Environmental Research Center (SERC; Fig A in S1 File), Water Quality Monitoring Network (http://serc.fiu.edu/wqmnetwork/) [49]. Total nitrogen, total phosphate, and salinity were converted to raster files by interpolating the values at geographical coordinates using the inverse distance weighted approach in R [50]. The raster files were then resampled using the largest extent and cropped to the critical habitat area of *Acropora palmata* for analysis [51] based on reported acroporid observations.

Daily wind speed (m s^-1^) and wind-direction (degrees) data [52] were obtained to calculate fetch (kJ m^-2^) (i.e., the unobstructed open ocean distance) using the ’*fetchR*’ package in R [53]. We considered fully developed seas as > 38 km of open ocean. Both light intensity (Photosynthetic Available Radiation, PAR) (mol m^−2^ s^−1^) and chlorophyll-a concentrations (mg m^-3^) were obtained from Moderate Resolution Imaging Spectroradiometer (MODIS) Aqua Level 3 and downloaded from the NASA Ocean Color site (http://oceancolor.gsfc.nasa.gov/) at a 4-km resolution. Sea-surface temperatures (SSTs) (°C) were obtained from the Multi-scale Ultra-high Resolution (MUR) SST analysis at 1-km resolution and downloaded from the Jet Propulsion Laboratory MUR MEaSUREs Project (https://dx.doi.org/10.5067/GHGMR-4FJ04) [54].

In combination, the 10 environmental variables used to determine suitable habitats for *A*. *palmata* along the Florida reef tract included: (1) fetch (kJ m^-2^), (2) maximum sea-surface temperatures (SSTs) (°C), (3) minimum sea-surface temperatures (SSTs) (°C), (4) mean chlorophyll-a concentrations (mg m^3^), (5) maximum light intensity (mol m^−2^ s^−1^), (6) minimum light intensity (mol m^−2^ s^−1^), (7) mean total nitrogen (N) (ppm), (8) mean total phosphorus (P) (ppm), (9) maximum salinity (ppt), and (10) minimum salinity (ppt) (Figs A and B in S1 File).

### Data analysis and model selection

Predictor variables were tested for multicollinearity via the ’*corrplot*’ package [55] and when correlations were apparent for related variables, one of the variables was dropped from the model based on relevance to *A*. *palmata* occurrence. When a site was marked as supporting the species, it remained ‘present’ at the site for the duration of the study. We used 80% of the data to train the models and 20% of the data to test the models using k-folding in R [50]. A suite of analytical approaches was used to analyze the data and models were compared using the Area Under the Curve (AUC) value [56]. The model resulting in the highest AUC score was a boosted-regression tree model with a tree complexity of 5, a learning rate of 0.005, and a bag fraction of 0.5 (AUC = 0.974). The optimal number of trees was determined using a cross-validation stagewise function [57]. Partial-dependency plots were generated to determine the relationships between the environmental variables and the occurrence of *Acropora palmata*. A probability of occurrence map for *A*. *palmata* was generated using the threshold method ‘*spec_sens’* (i.e., specificity-sensitivity threshold) [58] to establish a threshold value based on maximizing the sum of specificity (true negative) and sensitivity (true positive) values. The threshold method transformed the model predictions from probabilities to a binary score (i.e., presence = 1; absence = 0) based on the highest sum of true-positive and true-negative values.

## Results

**Fig 2:**
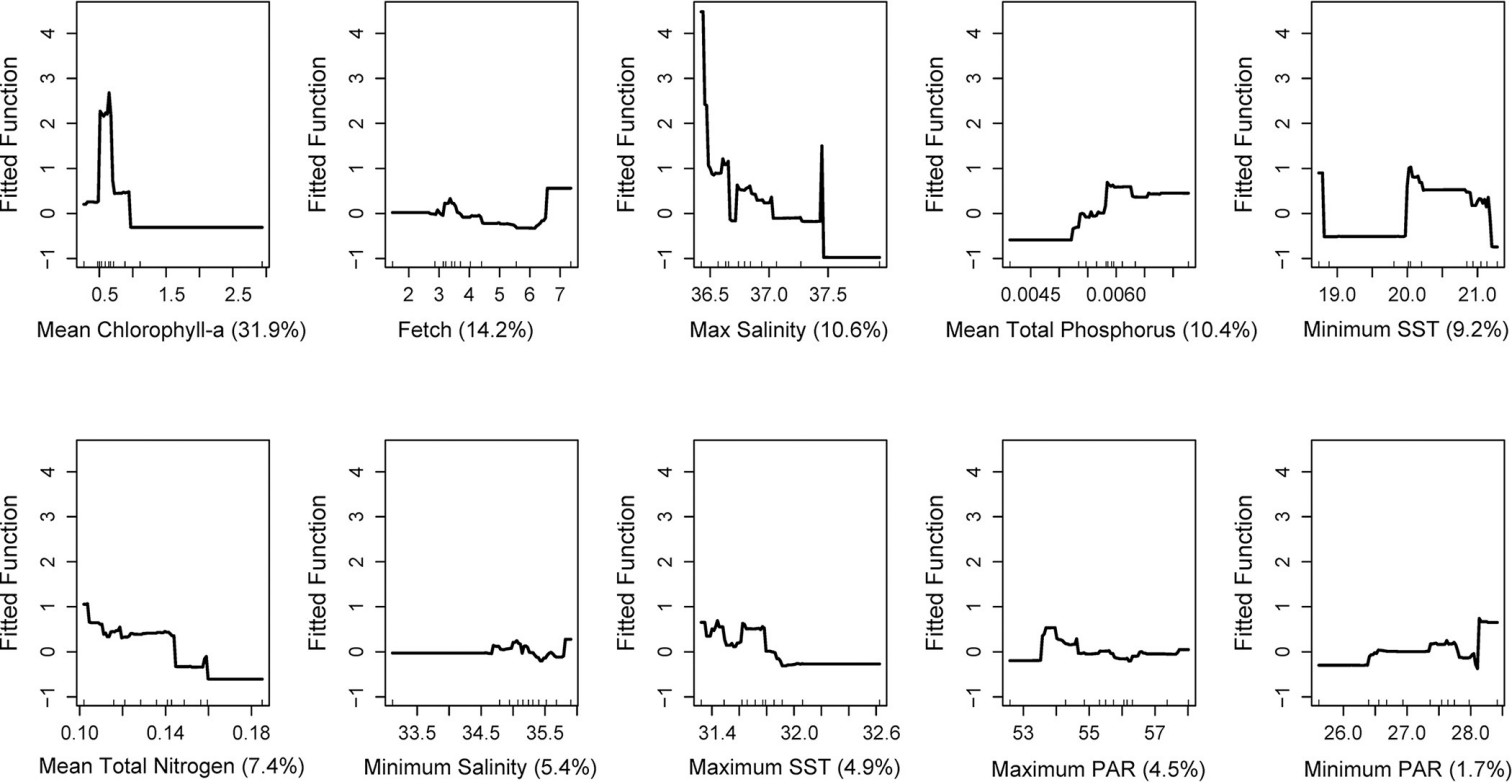
Partial-dependency plots of the relationships, expressed as a percentage, between 10 environmental variables and the presence of *Acropora palmata*, along the Florida reef tract, USA from 7,214 sites sampled between 1999 and 2019. The 10 environmental variables used to determine the suitable habitats (listed in order of predictive strength) included: (i) generally low mean chlorophyll-a concentrations (< 1 mg m^-3^), (ii) moderate fetch (3 kJ m^-2^), (iii) salinities between 20 and 37.5 ppt, (iv) temperatures between 20 and 32°C, (vi) low mean concentrations of total nitrogen (0.16 ppm), and (iv) irradiance between 26.5 and 53.5 mol m^−2^ s^−1^.

**Fig 3:**
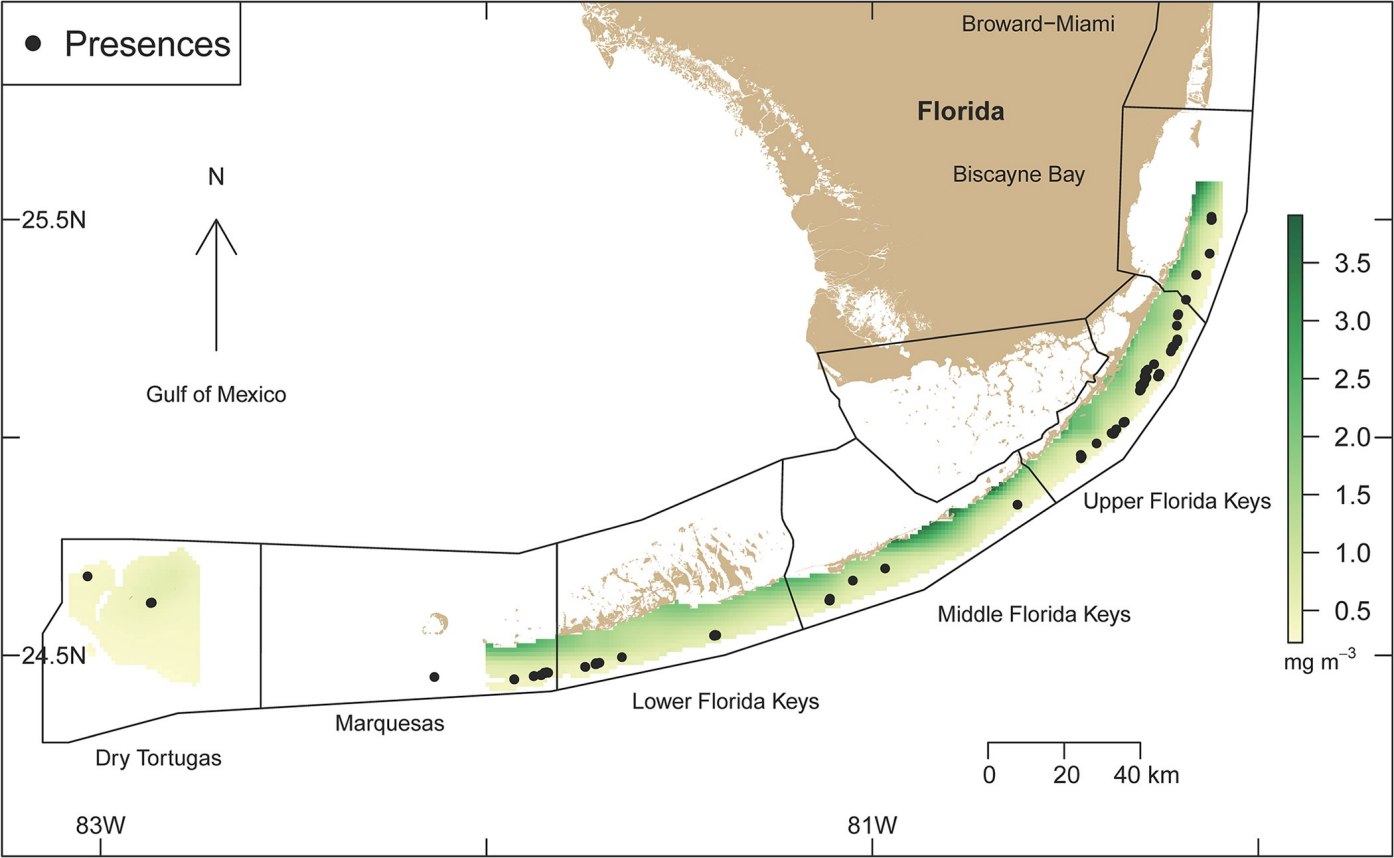
Mean chlorophyll-a concentrations from 1997 to 2021 and the presence of colonies of *Acropora palmata* between 1999 and 2019 along the Florida reef tract, USA.

Our boosted-regression-tree model for *Acropora palmata* predicted that mean chlorophyll-a concentration was the most important contributor in determining the distribution of *A*. *palmata* along the Florida reef tract. The results show that the species was less likely to occur in habitats with high average chlorophyll-a concentrations, > 1 mg m^-3^ (Figs 2 and 3). The occurrence of *A*. *palmata* was also most likely in habitats with moderate to high fetch of approximately 3 to 5 kJ m^-2^, in environments with salinities between 34.5 and 37.5, with sea-surface temperatures between 20°C and 32°C, and with light intensities between 26 and 54 mol m^-2^ s^-1^. Regarding nutrient concentrations, *A*. *palmata* was most likely to occupy habitats where total phosphorus was between 0.005 and 0.0065 ppm and with total nitrogen below 0.16 ppm.

**Fig 4:**
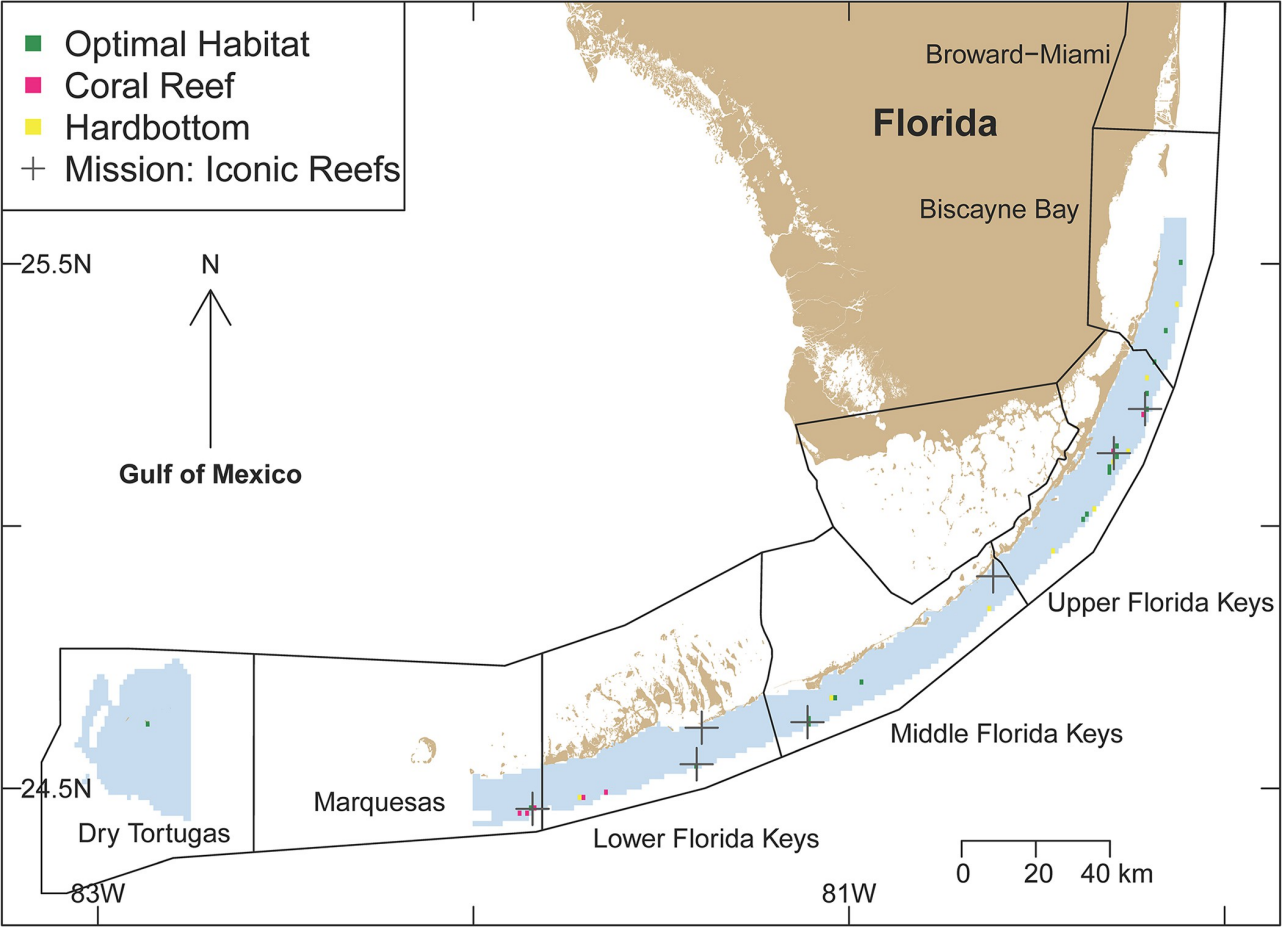
A map depicting present-day optimal locations, with suitable habitats and substrates identified in this study, for critically endangered *Acropora palmata* corals along the Florida reef tract, USA. The 10 environmental variables used to determine the suitable habitats (listed in order of predictive strength) included (1) mean chlorophyll-a concentrations (mg m^-3^), (2) fetch (kJ m^-2^), (3) maximum salinity (ppt), (4) mean total phosphorus (P) (ppm), (5) minimum sea-surface temperatures (SSTs) (°C), (6) mean total nitrogen (N) (ppm), (7) minimum salinity (ppt), (8) maximum sea-surface temperatures (SSTs) (°C), (9) maximum light intensity (mol m^−2^ s^−1^), and (10) minimum light intensity (mol m^−2^ s^−1^). The suitable habitats are depicted in green and the unsuitable habitats are depicted in blue. The suitable habitats overlaying suitable coral-reef and hard-bottom substrates are depicted in pink and yellow, respectively. Note: the seven Mission: Iconic Reefs, depicted as black crosses, are part of a 5-year coral-outplanting project by NOAA fisheries [48]—the names of which (from north to south) are Carysfort Reef, Horseshoe Reef, Cheeca Rocks, Sombrero Reef, Newfound Harbor Reef, Looe Key, and Eastern Dry Rocks.

Geographically, the most suitable locations for *A*. *palmata* were predicted to be in the upper and western-lower Florida Keys, Biscayne Bay, the eastern Marquesas, and the Dry Tortugas (Fig 4; for details also see S2–S4 Files, which are Google Earth kmz layers). However, differences in suitable habitats from nearshore to offshore were evident across these locations. For example, suitable habitat was apparent on outer reefs and mid-channel patch reefs in the upper Florida Keys but only on outer reefs in the lower Florida Keys. The only subregion with suitable nearshore habitat for *A*. *palmata* was in the upper Florida Keys, and although this nearshore habitat does not support substantial coral reef and hard-bottom substrate, it could potentially be suitable for the establishment of coral nurseries (Fig 4).

Coral reef and hard-bottom substrates (i.e., suitable habitats) that coincided with optimal environmental variables for *A*. *palmata* restoration were evident in the southern Biscayne Bay region, throughout the upper Florida Keys, the western-lower Florida Keys, the eastern Marquesas, and the Dry Tortugas. By contrast, minimal coral reef and hard-bottom substrate coincided with the optimal environmental variables for *A*. *palmata* restoration in the middle Florida Keys (Fig 4).

## Discussion

The present study identified optimal geographical locations for *Acropora palmata* colonies along the Florida reef tract. The least suitable habitats appear to be in the middle Florida Keys. The most suitable habitats appear to be in Biscayne Bay, the upper Florida Keys, the western-lower Florida Keys, the Marquesas, and the Dry Tortugas (Fig 4). Sites with suitable *A*. *palmata* habitat include potential restoration sites on reefs that had previously supported *A*. *palmata* but also include novel habitats and locations that did not historically support *A*. *palmata*.

Historically, *Acropora palmata* was known to inhabit shallow wave-exposed reef crests [1] but colonies of the species were also prevalent in shallow, sandy back-reef zones [59]. The locations that were predicted by the model as optimal for *A*. *palmata* but that did not support contemporary coral reefs or hard-bottom substrates may be suitable as potential nursery sites. Such potential nursery sites were evident throughout the Florida reef tract, including several mid-channel sites (Fig 4). *A*. *palmata* was found on shallow fore-and-back reefs in suitable habitats along the Florida reef tract, but it rarely occurred in high-nutrient environments, where chlorophyll-a concentrations were, on average, high. At a broad scale, the middle Florida Keys and the lower portion of the upper Florida Keys—two locations that fall directly in line with discharge from Florida Bay—consistently experience high levels of nutrient enrichment and productivity [60] and were identified as not offering suitable habitats for *A*. *palmata*. The middle Florida Keys appears to have been a consistently unsuitable habitat for *A*. *palmata* growth through the late Holocene period [1, 61]. The large channels in the middle Florida Keys expose reefs to inimical water quality, unsuitable for both coral growth and reef development [1, 62]. The source of unfavorable conditions appears to stem from Florida Bay, which experiences a broad range of temperature, salinity, nutrients, and turbidity over a short timescale [1, 62]. The broad range of such unfavorable environmental conditions stemming from Florida Bay has led to the underdevelopment of reefs in the middle Florida Keys, which began more than 6,000 years before present [63]. The middle Florida Keys has been recently dominated by non-reef-building corals and sparse boulder corals [61, 62]. These insights suggest that contemporary restoration in the Middle Keys should focus on coral species other than acroporids, because acroporids, historically, have been unable to survive a wide range of environmental conditions and high-nutrient concentrations.

While consistently high chlorophyll-a concentrations can be detrimental to corals, previous studies have also shown that corals in habitats with low chlorophyll-a concentrations and high irradiance were more prone to bleaching during thermal-stress events than corals in turbid, low-irradiance environments [64]. While our results show that maximum sea surface temperature was not the strongest predictor of *Acropora palmata* colonies in the past two decades (see Fig 2), our model does provide valuable insight into the potential effects of marine heatwaves. By using maximum and minimum values of temperature in previous decades as predictive variables to capture the niche space of *Acropora palmata*, we were able to predict that persistence would be low when sea surface temperatures exceeded 32°C (Fig 2). Indeed, water temperatures exceeded 32°C during the 2023 summer, causing extensive coral mortality in the Florida Keys (R.B. personal observations).

The identification of suitable locations for coral outplanting and nurseries is essential for restoring coral populations and conserving resources along the Florida reef tract. In 2019 Mission: Iconic Reefs was initiated to restore and conserve seven reefs along the Florida reef tract utilizing a comprehensive restoration approach [48]. In the Iconic Reefs implementation plan, the following targets of *A*. *palmata* outplants are planned per reef: (i) 77,613 at Carysfort Reef, (ii) 0 at Cheeca Rocks, (iii) 19,705 at Eastern Dry Rocks, (iv) 8,068 at Horseshoe Reef, (v) 19,534 at Looe Key, (vi) 0 at Newfound Harbor Reef, and (vii) 5,496 at Sombrero Reef (Fig 4), although targets may be adjusted over the course of the restoration. Our findings of optimal environmental localities support the Iconic Reefs implementation plan to avoid outplanting *A*. *palmata* on inshore patch reefs and middle Florida Keys sites, such as Newfound Harbor and Cheeca Rocks, where *A*. *palmata* have not historically occurred. Our findings also support the premise that suitable habitat for *A*. *palmata* occurs in the upper and lower regions of the Florida reef tract, such as Carysfort Reef and Eastern Dry Rocks, respectively. However, the range of suitable habitats surrounding Horseshoe Reef is supported by the extensive surrounding stands of *A*. *palmata* (not represented in surveys included in the model) [65], at least until the 2023 thermal-stress event reduced the population (R.B, personal observations). The moderate levels of *A*. *palmata* outplant targets in the Iconic Reefs implementation plan for Sombrero Reef and Looe Key is also supported by our results, as there are limited suitable habitats on these reefs.

Our study builds on the foundational work that showed the greatest differences in benthic composition [66] and outplant survival [47] were apparent at the reef scale (10–20 km). Such work suggests that since coral survival is not a random phenomenon, differences in conditions among reefs play a central role in recruitment potential and population persistence. For example, D’Antonio et al. [67] found that *A*. *palmata* is influenced by local topography and flow rates. Including high-resolution data on local topography, water-flow rates [68], local temperature anomalies, and high-resolution light conditions as predictive variables will improve the accuracy of future species-distribution models. Ideally, future research will also take a hierarchical approach by combining regional-scale conditions, such as in the present study, with local-scale influences and phenotypic diversity [69] to predict coral survival.

In conclusion, our study incorporated long-term, regional environmental data into a boosted-regression-tree model to identify optimal locations for critically endangered *A*. *palmata* corals along the Florida reef tract. This research facilitates site selection for areas of conservation, coral restoration, and coral nurseries, and supports initiatives such as Mission: Iconic Reefs to ensure that critically endangered corals such as *Acropora palmata* can persist on Florida reefs into the future.

## Supporting information

S1 File Details of the coral reef monitoring programs in Florida, further information on the environmental variables, and raster files for the seven environmental variables. (DOC)

S2 File Geographical locations of optimal habitat for *Acropora palmata* survival in the form of Google Earth kmz layer. (ZIP)

S3 File Geographical locations of optimal hardbottom habitat for *Acropora palmata* survival in the form of Google Earth kmz layer. (ZIP)

S4 File Geographical locations of optimal coral-reef habitat for *Acropora palmata* survival in the form of Google Earth kmz layer. (ZIP)
