# Supplementary material for: Environmental predictors for the restoration of a critically endangered coral, Acropora palmata, along the Florida reef tract
Source: PLoS One. 2024 Jan 2;19(1):e0296485. doi: 10.1371/journal.pone.0296485 (PMC10760844; doi:10.1371/journal.pone.0296485)
Supplement: S1 File — (DOC) [file pone.0296485.s001.doc]

**Supplementary Document**

**Environmental predictors for the restoration of a critically endangered coral, *Acropora palmata,* along the Florida reef tract**

Raymond B. Banister^1^, T. Shay Viehman^2^, Stephanie Schopmeyer^3^, Robert van Woesik^1*^

^1^ Institute for Global Ecology, Florida Institute of Technology, Melbourne, FL, 32901 USA.

^2^ National Centers for Coastal Ocean Science, National Ocean Service, National Oceanic and Atmospheric Administration, Beaufort, NC, 28516, USA.

^3^ Florida Fish and Wildlife, Fish and Wildlife Research Initiative, 100 8th Avenue SE, St. Petersburg, FL, 33701, USA.

* Corresponding author

Email: [rvw@fit.edu](mailto:rvw@fit.edu)

Table A. Details of coral monitoring programs in Florida with *Acropora palmata* data used in the species-distribution models. *A.* palmata was considered ‘present’ at a site for the model input if it was present for any of the datasets or years.

| **Program name** | **Program organization** | **Methods and survey area** | **Years of survey** | **Survey design** | **Total numbers of sites/n presence/n absence used in *Acropora palmata* models** | **Website and references** |
| --- | --- | --- | --- | --- | --- | --- |
| Coral Reef Evaluation and Monitoring Project (CREMP) | Florida Fish and Wildlife Conservation Commission (FWC) | 4 22 m^2^ belt transects per site | 1999–present; annual | 38 fixed sites (113 stations) in Keys; 3 fixed sites (20 stations) in Dry Tortugas | 5,994 total  1,351 Presences  4,643 Absences | https://myfwc.com/research/habitat/coral/cremp/ |
| Disturbance response monitoring | FWC | 2 10 m^2^ belt transects per site | 2005–present; annual | Stratified random sites to 20 m depth. | 7,177 total  160 Presences  7,017 Absences | <https://myfwc.com/research/habitat/coral/drm/>  [1,2] |
| *Acropora* specific monitoring | National Oceanic and Atmospheric Administration (NOAA) and FWC | 3-4 7m-radius plots per site in Keys and SE FL; 2 7m-radius plots in Dry Tortugas | 2011-present; quarterly in Keys and SE FL, and three times a year in Dry Tortugas | 7 fixed sites in Keys and SE FL; 1 site in Dry Tortugas | 3,182 total  3,182 Presences  0 Absences | FWC- unpublished data |
| National Coral Reef Monitoring Program (NCRMP) | NOAA | 1 10 m^2^ belt transect/site | 2014–present; biennial | Stratified random sites to 30 m depth | 1,098 total  8 Presences  1,090 Absences | https://www.coris.noaa.gov/monitoring/  [3] |
| Sanctuary Coral Reef Ecosystem Assessment and Monitoring Program (SCREAM) | Nova Southeastern University/ University of North Carolina Wilmington | 2 10 m^2^ belt transects per site | 1999–2011; annual/variable | Stratified random sites to 30 m depth | 8,046 total  2,569 Presences  5,477 Absences | [2] |


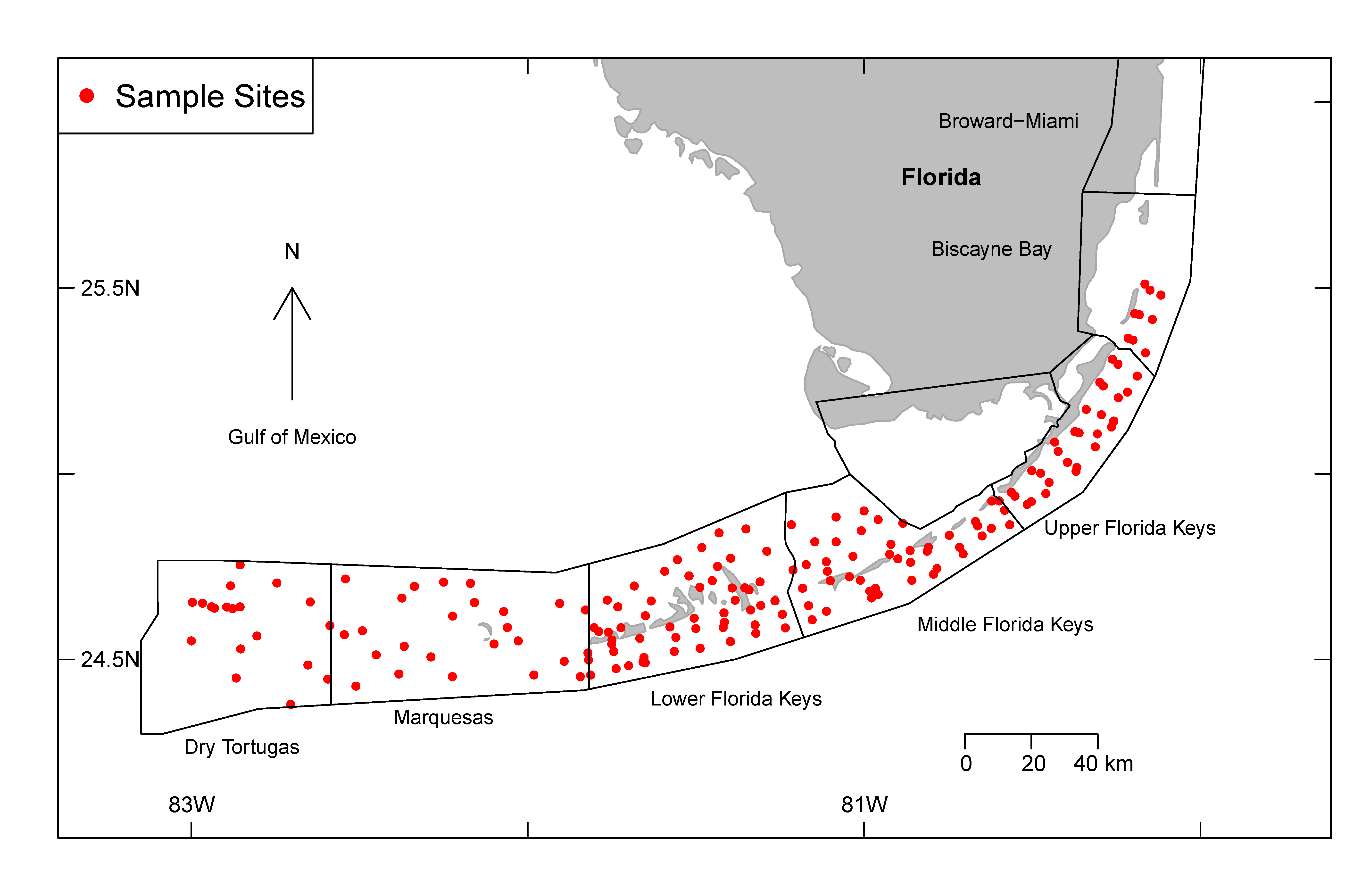


**Figure A.** SERC quarterly sampling locations (red) along the Florida reef tract [4].


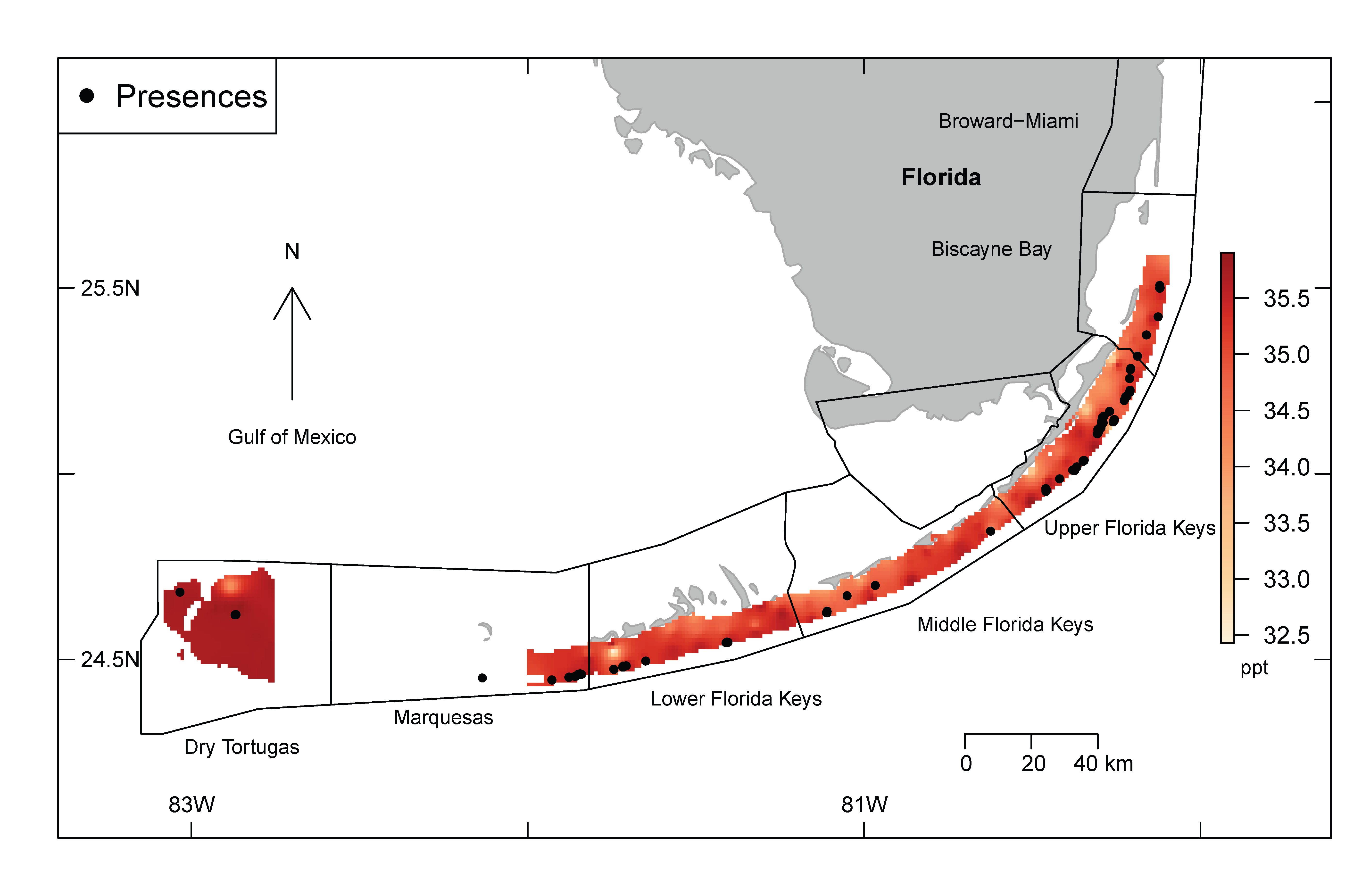

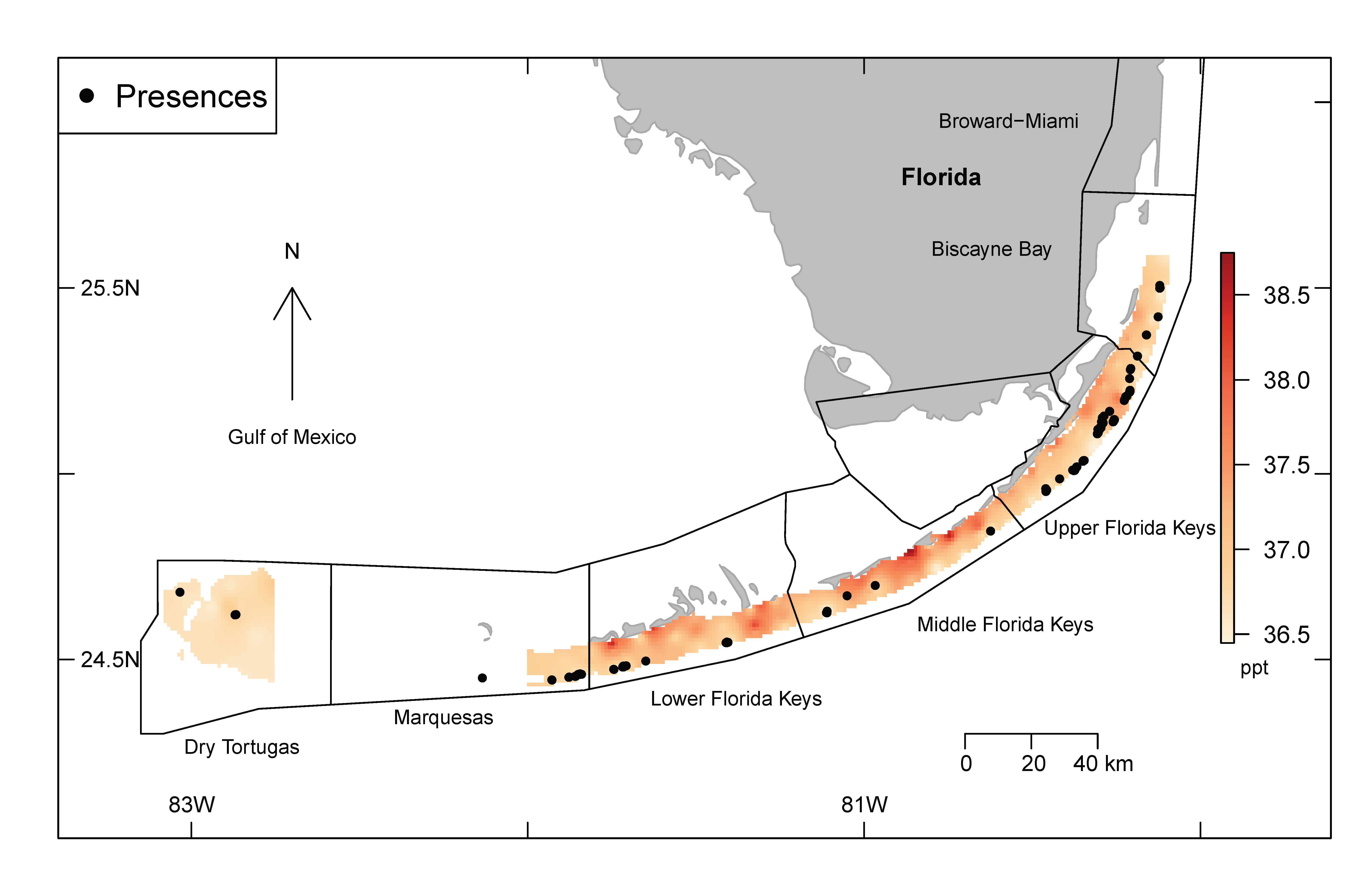

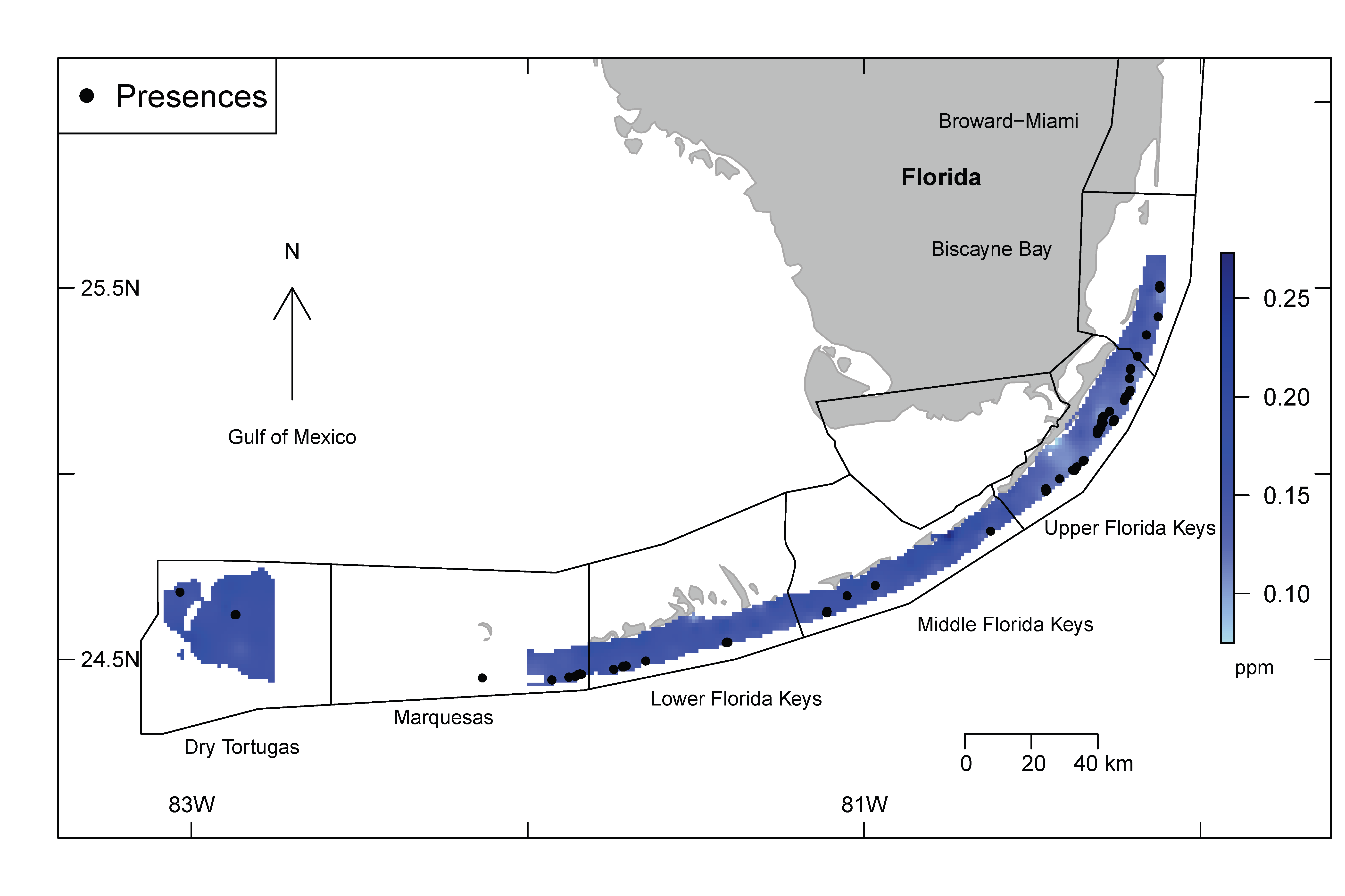

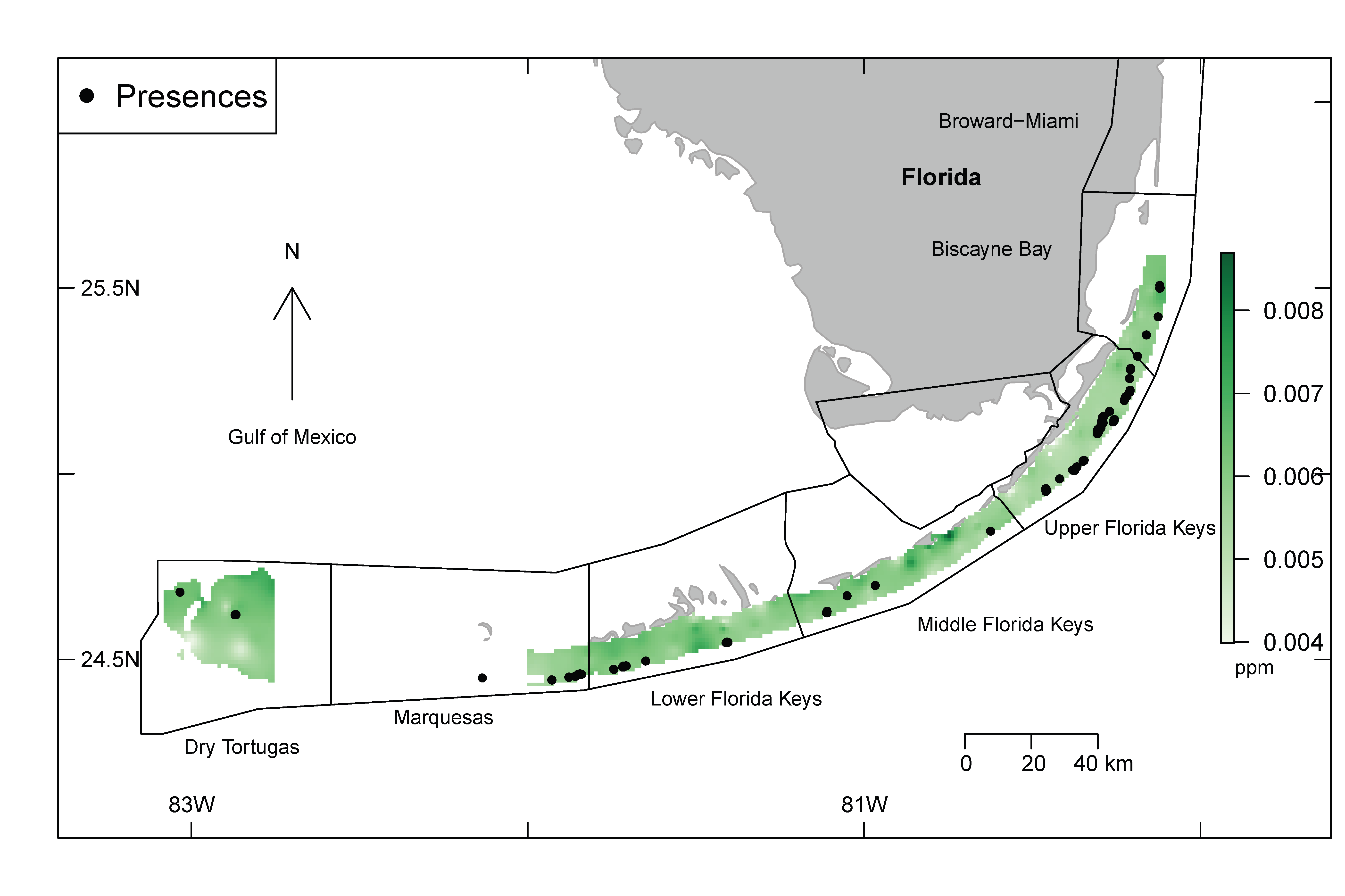


Mean Total Nitrogen

(b) Mean Total Phosphorus

(c) Minimum Salinity

(d) Maximum Salinity

**Figure B.** Rasters created from the Southeast Environmental Research Center, Water Quality Monitoring Network [4] database using an inverse distance weighted approach. Variables for near-substrate (a) mean total nitrogen (ppm), (b) mean total phosphorus (ppm), (c) minimum salinity (ppt), and (d) maximum salinity (ppt) are shown at 1-km resolution.


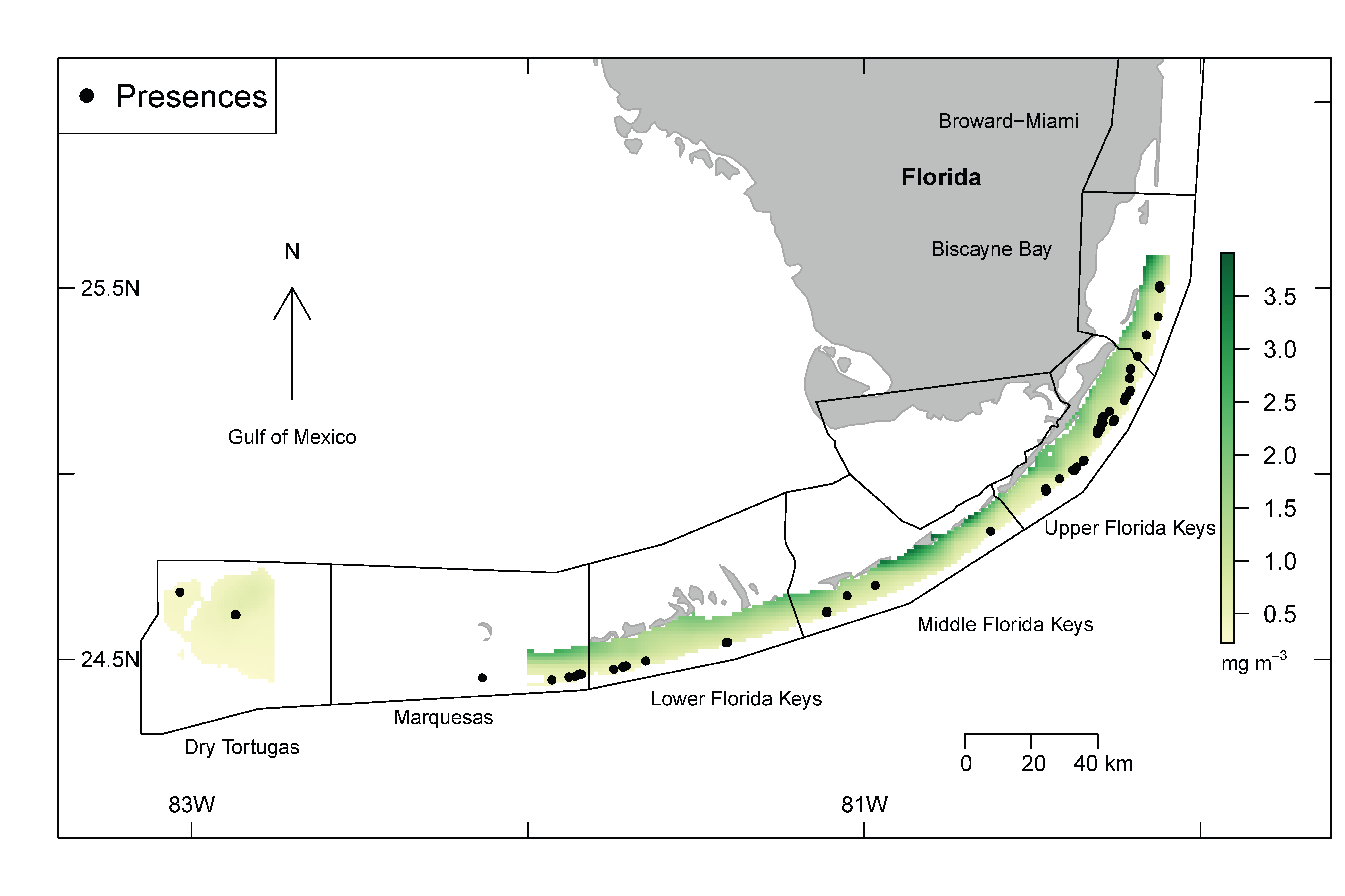

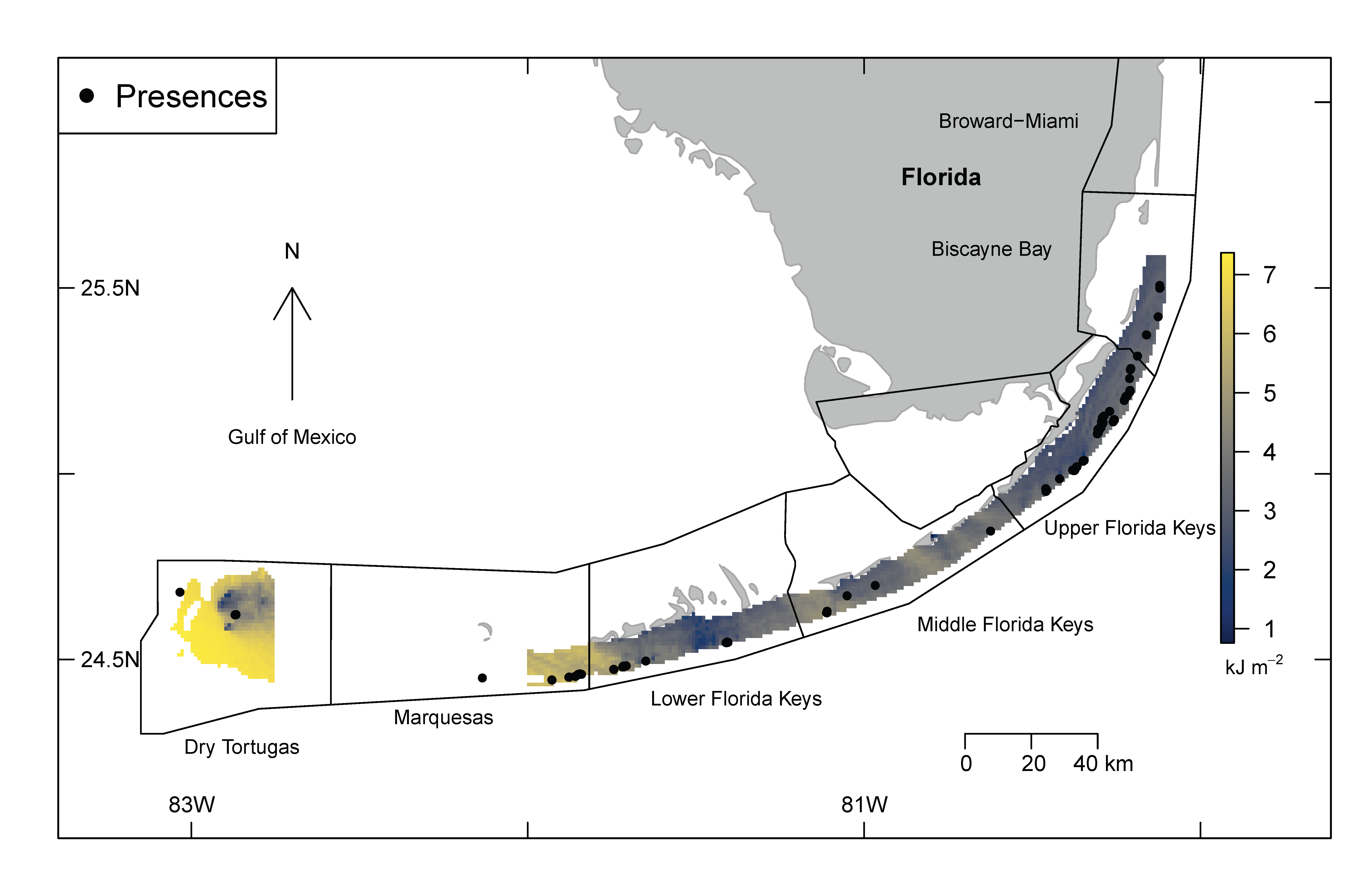

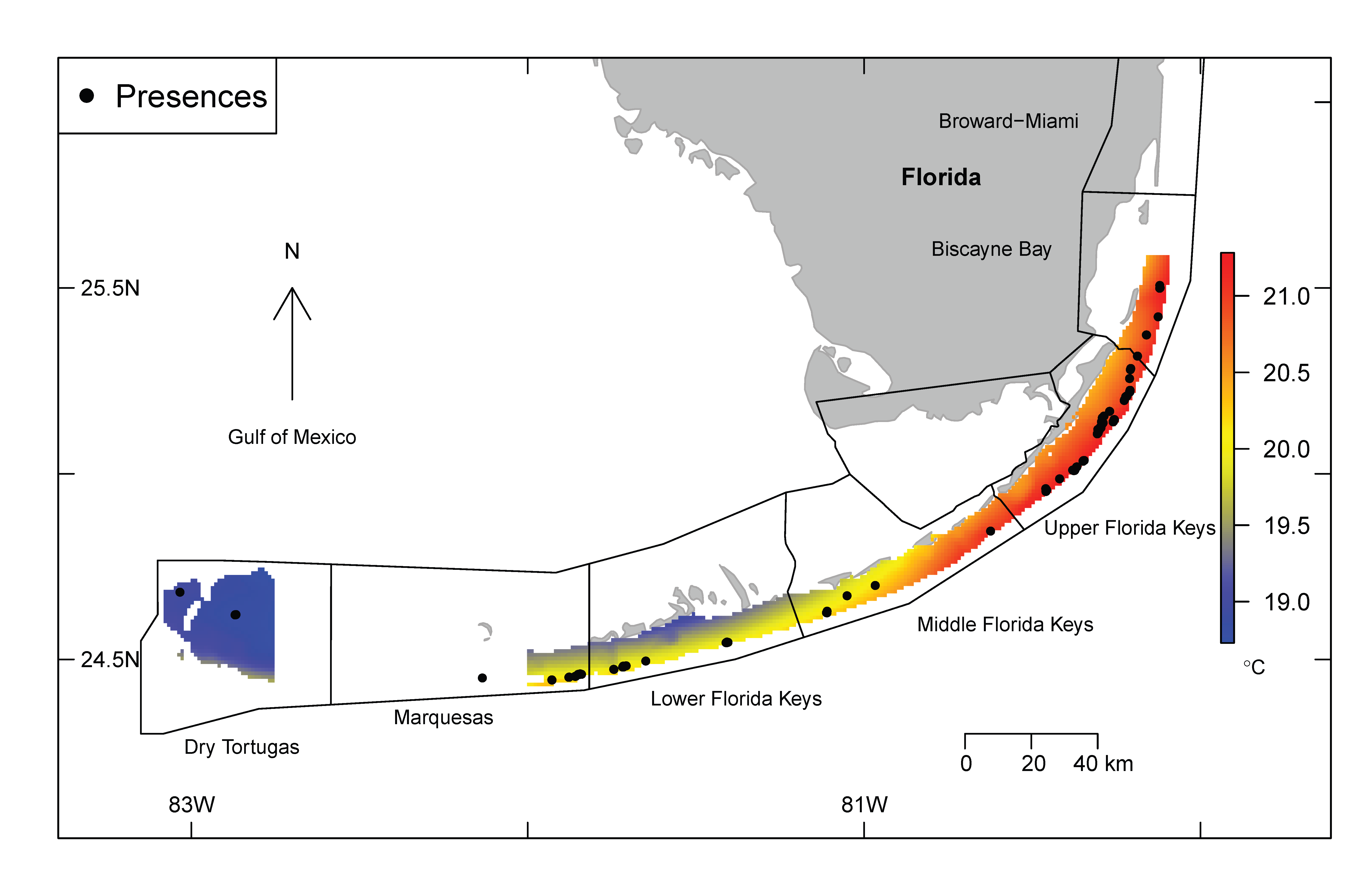

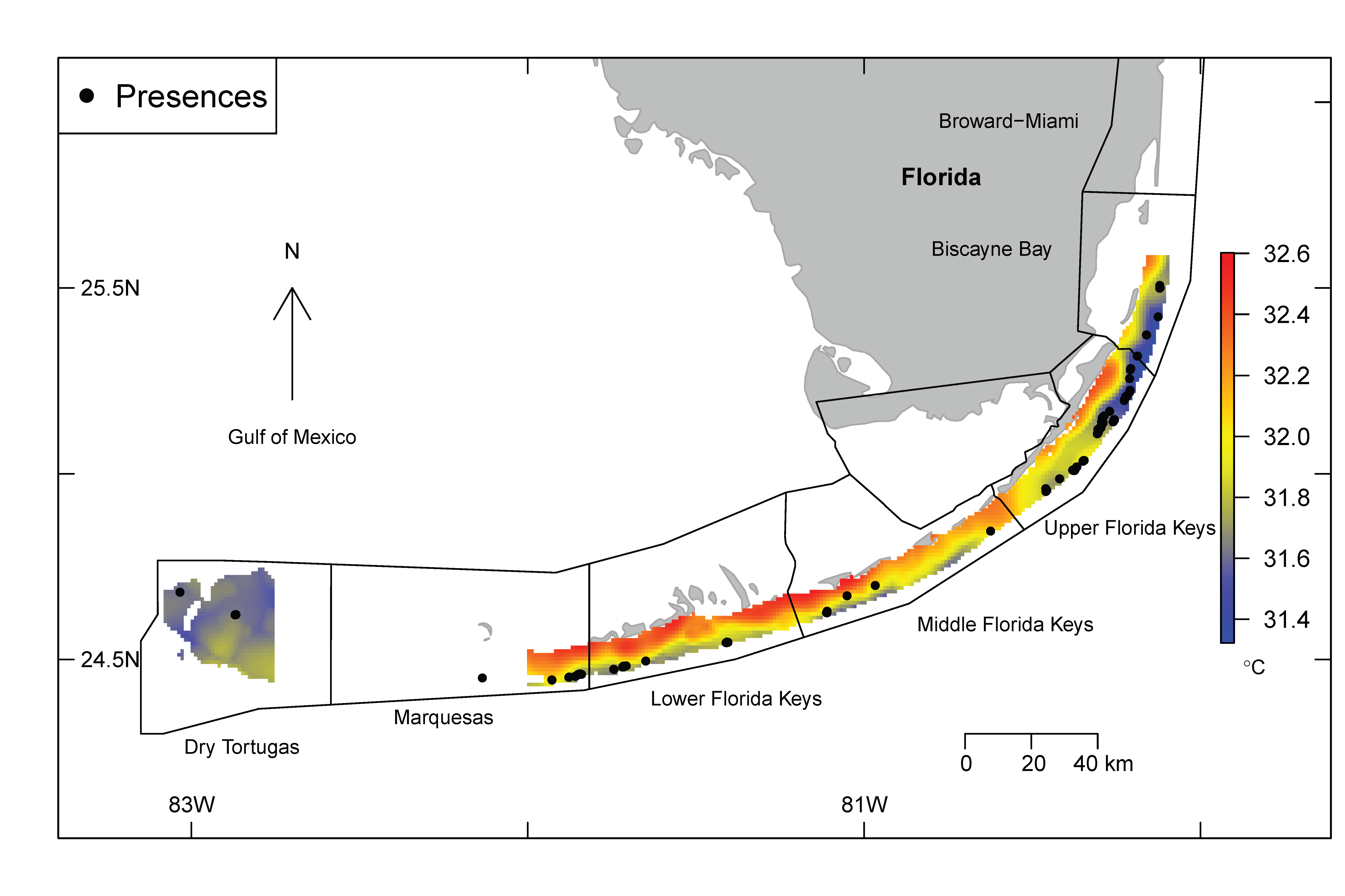

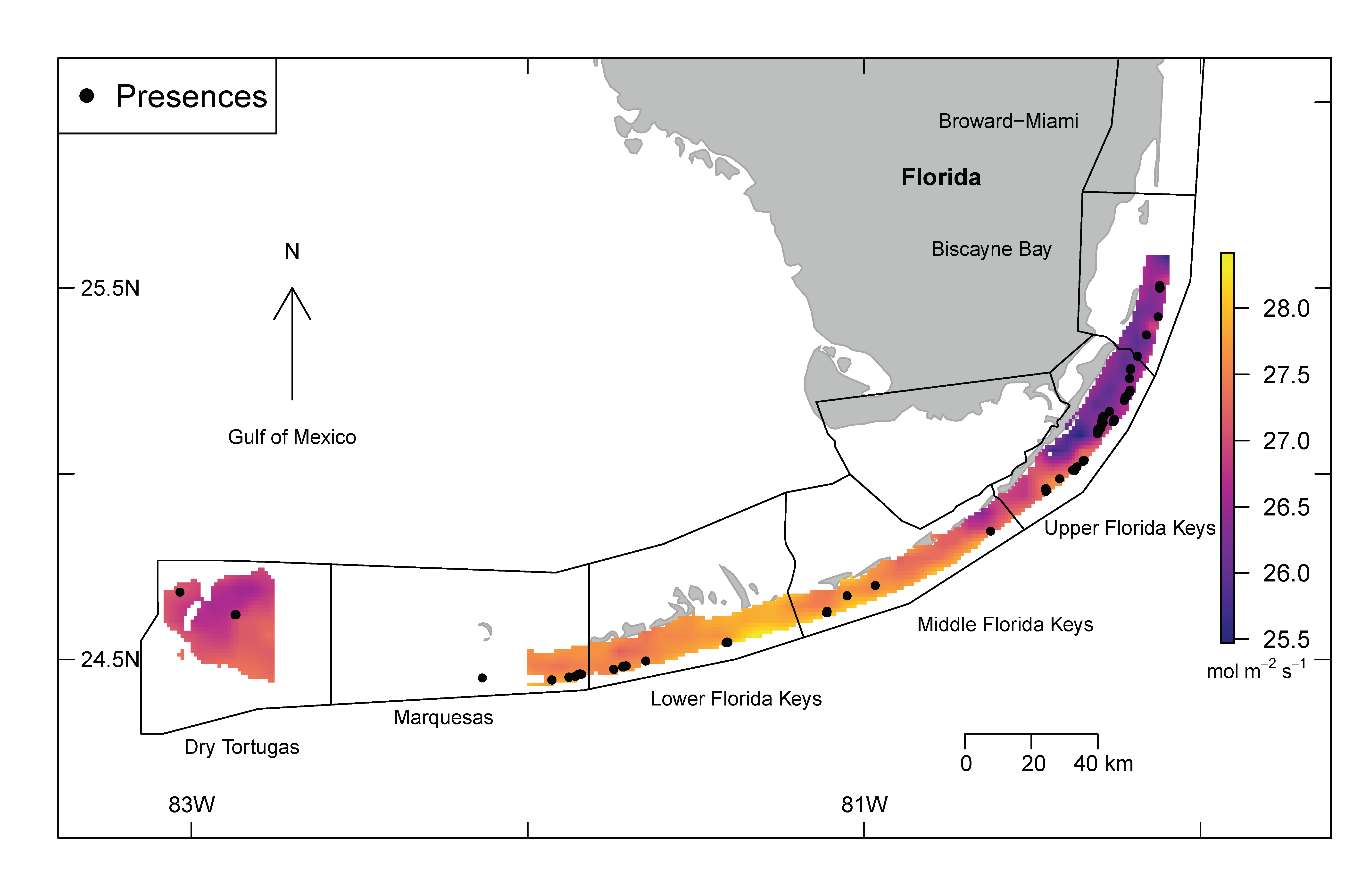

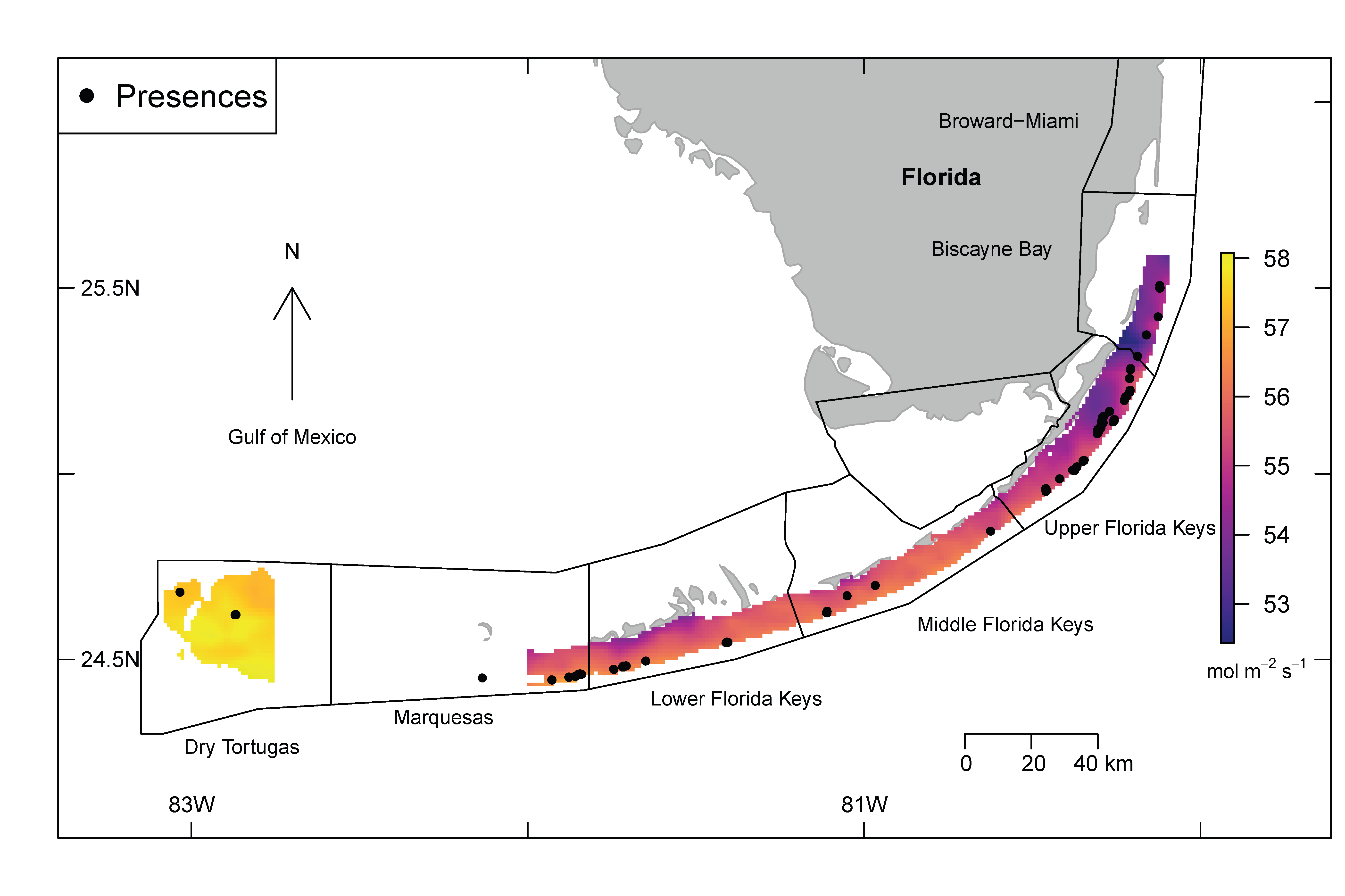


1. Mean Chlorophyll-a

(b) Fetch (km)

(c) Minimum SST

(d) Maximum SST

(e) Minimum Light Intensity

(f) Maximum Light Intensity

**Figure C.** Rasters showing the (a) mean chlorophyll-a concentrations (mg m^-3^), (b) fetch (kJ m^-2^), (c) minimum sea surface temperature (^o^C), (d) maximum sea surface temperature (^o^C), (e) minimum light intensity (mol m^−2^ s^−1^), and (f) maximum light intensity (mol m^−2^ s^−1^) at 1 km resolution.

**References**

1. Ault JS, Smith SG, Bohnsack JA, Luo J, Harper DE, McClellan DB. Building sustainable fisheries in Florida’s coral reef ecosystem: positive signs in the Dry Tortugas. Bull Mar Sci. 2006;78: 633–654.
2. Smith SG, Swanson DW, Chiappone M, Miller SL, Ault JS. Probability sampling of stony coral populations in the Florida Keys. Environmental Monitoring and Assessment. 2011b;183: 121–138. <https://doi.org/10.1007/s10661-011-1912-2>
3. Viehman TS, Groves SH, Grove LJW, Smith SG, Mudge L, Donovan C, et al. A quantitative assessment of the status of benthic communities on US Atlantic coral reefs using a novel standardized approach. 2023 [cited 17 Jul 2023]. doi:10.5343/bms.2022.0025
4. Boyer JN, Briceño HO. South Florida coastal water quality monitoring network. FY2006 Cumulative Report South Florida Water Management District, Southeast Environmental Research Center, Florida International University. 2007. Available: http://www.serc.fiu.edu/wqmnetwork./Report%20Archive/2006_CWQMN.pdf
